# Medical History from the Earliest Times

**Published:** 1892-08-20

**Authors:** E. T. Withington


### MEDICAL HISTORY FROM THE EARLIEST TIMES.

By E. T. Withington, M.A., M.B. Oxon.

### XXII.—OFFICIAL MEDICINE IN ANCIENT ROME.

When the Emperor Claudius made a speech to the Senate requesting exemption from taxes for the island of Cos, he of course introduced the name of Æsculapius, and he told his hearers that his own medical attendant, Xenophon, was a direct descendant of that divine physician. If the Emperor did not make this up for the occasion, Xenophon is the latest known member of the great family of the Asclepiadæ; he is also, perhaps, the first of a new order, that of the Imperial Archiatri, but he seems to have gained this honour by breaking the oath of his guild and assisting in the murder of his master. Physicians, alas! are too often found as abettors in the crimes of the early Empire, but we must remember that many were still in the position of slaves, or, at best, freedmen. In the hands of an Agrippina or a Messalina the choice must often have been between virtue and life, and we need not greatly blame a pagan practitioner if he sometimes preferred the latter. Whatever else is known of Xenophon is to his credit. We have the inscriptions of the people of Cos praising him as their public benefactor; we have his own epitaph, which relates his long service with the army, in which he gained the blunt spear and the gold crown, rewards for distinguished conduct in the field; and we may hope that that one terrible crime is the only blot on the otherwise honourable life of the last Asclepiad.

On his tombstone Xenophon is entitled "Archiatrus," or chief physician, to "the august divinities," a term probably meant to include Agrippina and Nero; and he was succeeded in that office by Andromachus, inventor of a famous theriac, or antidote. It used to be thought that the Archiatrate was a purely Roman institution, but the office, as well as the name, appears to be Greek, for the title is applied in inscriptions to the physicians of Eastern kings, such as Mithridates of Pontus. We may, perhaps, even trace its origin to Egypt, where the title, "chief physician," was very common, and was, as we have seen, applied to the most ancient known practitioner of medicine. But whatever be its antiquity, the name has survived, in an altered form, to our own day, for philologists tell us that the German "arzt" is derived, not from artifex, but from archiater, which became corrupted to "ærsater."

At Rome the office underwent a rapid and remarkable development, and diligent historians distinguish no less than five classes of Archiatri. Of these, the first may be considered a natural product of the gradual conversion of the Republican "imperator" into an Oriental monarch. The sacred person of the Emperor could only be tended by physicians themselves marked off from the common herd, and at the Court of Constantinople the "Archiatri palatini," or "sacri palatii," formed a distinct body, whose president had the title of Count, and who themselves ranked among the "perfectissimi," and might sometimes supply even an imperial vicar or a proconsul of Africa.

The second class were a further development of those communal physicians who had so long existed in Greek cities. The privileges which the gratitude of Augustus had bestowed on the whole profession were restricted by Antoninus to five, seven, or ten physicians in each city, according to its size, who were elected by the municipality and neighbouring landowners, and who, in the reign of Constantine, had acquired the title of "Archiatri populares."

At Rome the need of a public medical service seems to have been less obvious, and it was not till A.D. 378 that an Archiater was appointed for each of the fourteen districts of the city by a decree of the Emperor Valentinian. These differed from the former class in being under the control, not of the local authority, but of the central government, while vacancies were filled up by the votes of the survivors, whose choice, perhaps, had to be approved by the Emperor.

The fourth and fifth classes comprised the heads of those clubs or societies, "Collegia" or "Scholæ," which sprang up in great numbers during the early Empire, both in the medical and in other professions, and the physicians of the "porticus xystus," or gymnasium, at Rome, and of the Vestal Virgins, who continued till the beginning of the fifth century.

The Archiatri populares were permitted to practise privately, but their chief duties were to attend the sick poor, and to instruct pupils in their art. The Imperial edicts command the greatest care to be taken in their election, which was preceded by some sort of examination, but they do not seem to have had any control over the profession generally, and, as in ancient Greece, the practice of medicine was open to anyone. The institution, however, doubtless had a favourable influence on medical education. Those who intended to aspire to the Archiatrate would be specially careful as to their course of study, of which they might have to give an account; while at the same time the number of well-qualified teachers was greatly increased, and schools of medicine arose in various localities, some of which, as at Beneventum and Avranches, lasted till the beginning of the Middle Ages. Finally, the acquirement of a legally recognised title, carrying with it many privileges and immunities, by large numbers of physicians tended in no small degree to raise the social status of the whole profession.

The epitaph of Valeria Verecunda, which calls her "head midwife of her district," seems to indicate that the poor of Rome were supplied with special as well as general medical aid, and that the ancients had already to some extent settled the question of the registration of midwives. That class, indeed, held a relatively much higher position than at present. Pliny speaks of a "Nobilitas obstetricum;" the Justinian code estimated the obstetrix of servile rank at the same high value as the "Servus medicus," and they are frequently alluded to by the general title, "Medicæ," for they by no means confined themselves to obstetrics, or even to the diseases of women and children. According to the satirists of the period, these "medicæ" were of anything but high character, and were ever ready to assist in poisonings, abortions, and all kinds of wickedness; but a fairer and not unfavourable estimate may be gathered from the writings of their colleagues of the other sex. Thus the great Heraclides of Tarentum dedicates his treatise on nose-bleeding to a certain Antiochis, some of whose prescriptions Galen himself has not disdained to copy, while at a later period Theodore Priscian addressed the gynæcological part of his work on medicine to Victoria, whom he calls "the sweet servant of my art." The Roman law directed that their assistance should be invoked for the decision of certain medico-legal questions, a custom which was probably the origin of the now nearly extinct "jury of matrons."

In conclusion, space only permits a brief notice of the medical rubbers, the "iatroliptæ" of the Greeks, who flourished greatly at Rome, and whose reputation was even worse than that of the medicae and the drug-sellers. Their depravity may be best expressed in the language of the philosopher Seneca, who declares that he would rather thrust his band into the fire, like Mucius Scaevola, than have it massaged by any one of them, male or female. Pliny the younger, however, was less fastidious, or more fortunate, for we find him requesting the Emperor Trajan to grant the citizenship not only to his physician, Marinus, but also to his private rubber, Harpocras.

